# An exploration of person-centred approach in end-of-life care policies in England and Japan

**DOI:** 10.1186/s12904-022-00965-w

**Published:** 2022-05-11

**Authors:** Chao Fang, Miho Tanaka

**Affiliations:** 1 Department of Social and Policy Sciences and Centre for Death and Society, University of Bath, Bath, BA2 7AY UK; 2 Japan Medical Association Research Institute, 2-28-16 Honkomagome, Bunkyo-ku, Tokyo, 113-8621 Japan; 3 Graduate School of Core Ethics and Frontier Sciences, Ritsumeikan University, 56-1 Tojiin Kitamachi, Kita Ward 603-8577 Kyoto, Japan

**Keywords:** End-of-life care, Policy, Person-centred approach, Best interests, Relationality, Existential distress, England, Japan

## Abstract

**Background:**

Increasing evidence has suggested that a person-centred approach (PCA) is beneficial not only for improving care outcomes but also for mitigating the pressure on public health systems. However, policy implementation gaps have prevented the translation of this complex framework into useful practical, ethical and moral stances for end-of-life care (EOLC). This article aims to explore the meaning and implications of person-centredness in EOLC policy discourses.

**Methods:**

By perceiving policy documents as a medium embodied with socio-political and cultural norms, we analysed how PCA in EOLC is constructed within specific socio-cultural contexts and the implications of these contexts on resultant care. Focusing on England and Japan, we conducted a critical policy analysis to examine and compare key policy and legal documents released between 2000 and 2019 in these two post-industrial and socio-culturally distinctive countries.

**Results:**

Our analysis found that the PCA is mobilised in policy discourses primarily through three interconnected dimensions: individual, relational and existential. While acknowledging that both countries have developed varied policy and legal mechanisms to emphasise holistic and integrated care with respect to these three dimensions, we also identified significant gaps in the pol icies both within and between England and Japan. They include ambiguity in defining patients’ best interests, fragmented support for social and family care and the neglect of existential needs.

**Conclusions:**

This cross-cultural analysis has revealed the complex nature of discourses around PCA in English and Japanese EOLC policies, which often concentrate on the multifaceted aspects of experiences as one approaches the end of life. Despite this, we argue that a more holistic construction of PCA is needed in EOLC policies not only in England and Japan but also more broadly, to encapsulate the richness of end-of-life experiences.

## Introduction

The person-centred approach (PCA) is at the forefront of end-of-life care (EOLC), aiming to prioritise the individual needs and values of patients and their families in decision-making, planning and resultant health and social care [1, 2]. Evidence suggests that this approach is beneficial for both improving outcomes and reducing care expenditure [3]. Given more people are going through a chronic process of complex symptoms when dying, how to deliver person-centred care in EOLC has increasingly become a public health interest. Hence, public policy seeks to mitigate the conflicts between the increasing demands of individual EOLC and the pressure on health and social welfare systems [4].

While a growing number of countries and regions have developed (or are in the process of developing) dedicated EOLC policies, a significant ‘policy-implementation gap’ remains [5]. This gap questions the inadequate processes of policymaking and unequal access to EOLC services. This gap is likely to be further amplified during the translation of PCA into practical, ethical and moral protocols. As a key principle, PCA has been extensively integrated into EOLC policy discourses, including policies, laws, and (inter) national organisational and occupational guidelines. However, PCA in EOLC policies potentially confront the competing agendas of prioritising individual care and public health interests, suggesting that policies may apply standardised lens to EOLC with limited emphasis on individuals and their holistic needs [6]. Therefore, it is imperative to examine how EOLC policies interpret and implement PCA and how these can interconnect with the real world.

This study focused on England and Japan to examine how PCA is embedded in their key EOLC policies and the implications of these policies on EOLC experiences across various settings. Despite being socio-culturally different, England and Japan are both affluent societies with highly developed care systems, that are also confronted with rapid population ageing and high degrees of medicalisation of dying [7]. Thus, both countries confront shared challenges, including increasing monetary input and structural reforms into health and social care for their ageing populations, EOLC and dementia support, as well as continuing debates on the legitimacy of euthanasia and assisted dying. Meanwhile, cultural divergences between individualism in England and family-centred values in Japan have shaped different and sometimes competing expectations and perceptions of EOLC [8, 9]. Thus, while having mature systems and multifaceted provision of EOLC, both countries have developed unique policy approaches to conform to their socio-cultural dynamics. To better understand PCA in EOLC policies, we critically examined key policy and legal documents from England and Japan, seeking to capture a broader picture of (1) how PCA is constructed in English and Japanese EOLC policy discourses, (2) the associated policy implementation gaps. Ultimately, this study aimed to identify avenues to strengthen EOLC policies in each country and further facilitate mutual learning.

### Understanding person-centred approach in end-of-life care

The concept of person-centredness has increasingly gained popularity in health and social care since the 1990s [1]. Central to this concept is a western emphasis on free will, asserting that care recipients are inherently self-sufficient and reflexive in making the right choice [10]. This conceptual focus has been widely operationalised as a person-centred approach (PCA), while various interchangeable terms (e.g., individual-, patient-, client-centred) have also emerged, emphasising the values and needs of patients as independent beings in different care/service contexts [11, 12]. More inter-personal PCA models have also been argued for to highlight the relational nature of individual needs and agency [2, 11]. Despite PCA being broadly adopted as an ethical principle in health and social care, there are diverse and even competing interpretations of what it means to be a person in different disciplinary and cultural contexts [2, 13, 14]. Thus, the conceptual connotations of PCA remain largely ambiguous and may lead to inadequate translation of person-centredness into practical and organisational spheres of care.

This ambiguity surrounding PCA is further compounded by complex and distressing circumstances in EOLC [12]. While emphasising patients’ dignity, autonomy and relationality during the dying process, PCA often confronts challenges for extending the person-centred principles past life to ensure a good death [15]. Prioritising PCA may also cause controversies in issues pertaining to euthanasia and assisted dying, contesting the boundaries of individual autonomy [16]. In light of these challenges, distinctive conceptual developments and practical innovations have evolved to respond to the complexity of PCA in EOLC [11, 12]. A conceptual maturing of person-centredness has occurred in the Hospice Movement, emphasising dying patients’ ‘total pain’ in response to their progressive and multifaceted deteriorations [17]. Building on this hospice philosophy, PCA has gradually been integrated into EOLC to promote more holistic and humanistic care for dying patients and their families [14]. In addition to care, advance care planning (ACP) has consistently emphasised PCA to enable people to take control of their care before losing the ability to make their own decisions [18].

PCA has also drawn criticism for not fully addressing the rich matrix of human experiences that encompass individual, familial, sociocultural and existential aspects of EOLC experiences [15, 19, 20]. By prioritising individual autonomy, PCA may inadequately address the relational and social nature of EOLC experiences, in which patient care and decision-making are often co-constructed by family and practitioners [2, 21]. PCA, which primarily focuses on medical settings, may also underestimate the role of community-based support, albeit there being a significant portion of people dying at home or in care facilities [3]. Furthermore, the western-centric PCA may inadequately recognise the values and needs of dying people from non-western cultures [21, 22]. Critics have also challenged the scope of PCA in response to patients’ deeper needs to preserve their social and spiritual being and to alleviate distress due to existential loneliness and diminishing personhood [20].

The complex and ambiguous nature of PCA can be particularly prevalent in EOLC policy discourses, as the standardised policy lens often does not focus on diverse needs of patients as unique beings [23]. Nonetheless, how policies define PCA in EOLC and how these definitions impact practice remain missing in extant literature.

## Methods

To allow for a nuanced understanding of the complexities behind the development and impact of EOLC policies, we adopted a critical policy analysis lens to examine how PCA is mobilised in EOLC policies within the unique contexts of England and Japan. By perceiving policies as a complex of discourses infused with socio-political values, practical interests and the needs of civilians, we sought to approach EOLC policies not as static and purely objective entities (e.g., natural laws), but as socially constructed artefacts which are constantly (re) shaped by the dynamic functioning of society [24]. Meanwhile, these collectively negotiated discourses can also mediate practice in reality [25]. To understand how policies have (or have not) reflected practice and how they have evolved in response to changing realities, we explored how PCA in EOLC as a ‘policy problem’ is constructed (both textually and discursively) within socio-cultural contexts and with respect to its implications on care. As such, our analysis is critical in a sense that we examined the EOLC policies in conjunction with the dynamics of EOLC practice, to uncover policy gaps in recognising and supporting PCA in EOLC for future improvement.

### Data collection

Given its focus on contesting policy as ‘something to be critiqued or troubled rather than accepted at face value’, critical policy analysis pays particular attention to the researchers’ theoretical perspectives and how the collection and examination of policy data are imbued with these understandings [25]. This theory-driven approach was deeply embedded in the process of identifying and selecting policy data in our study. Taking a social constructivist perspective (as explained above), we sought to critically understand how policies in England and Japan have been developed and have evolved to reflect the essential (and often changing) needs and values of PCA in different EOLC settings. As such, we intended to identify a series of influential policies that have shaped the national direction of English and Japanese EOLC development.

We aimed to identify official documents in England and Japan that lay out national policies and measures planned and developed by a governmental or legislative authority to achieve certain objectives regarding EOLC within the scope of the duties under their jurisdiction. We searched government databases and websites (as listed in Table 1) between August and December 2019, including GOV.UK (particularly the Department of Health and Social Care) in England and e-gov (particularly the Ministry of Health, Labour and Welfare) in Japan. The search terms included ‘end of life care’, ‘palliative care’ and ‘hospice care’ for England and ‘終末期医療’ (‘end of life care’ in Japanese), ‘緩和ケア’ (‘palliative care’ in Japanese), ‘介護’ (‘care/nursing’ in Japanese) for Japan. The selection parameters for our searches allowed for documents up until December 2019.

**Table 1:** Databases and websites for data collection

England	
Name of database or website	Link
UK GOV Policy Papers and Consultations Search Portal	https://www.gov.uk/search/policy-papers-and-consultations?content_store_document_type%5B%5D=policy_papers&order=updated-newest
Legislation.gov.uk	https://www.legislation.gov.uk/
Department of Health and Social Care Research and Statistics Search Portal	https://www.gov.uk/search/research-and-statistics?organisations%5B%5D=department-of-health-and-social-care&parent=department-of-health-and-social-care
Japan	
Name of database or website	Link
The Ministry of Health, Labour and Welfare Website	https://www.mhlw.go.jp/index.html (in Japanese)
Advisory panels and research groups related to the MHLW	https://www.mhlw.go.jp/stf/shingi/indexshingi.html (in Japanese)
List of the MHLW statistics	https://www.mhlw.go.jp/toukei/list/ (in Japanese)
e-GOV (operated by the Digital Agency)	https://elaws.e-gov.go.jp/ (in Japanese)
Japanese Law Translation Database System (operated by the Ministry of Justice)	http://www.japaneselawtranslation.go.jp/

Based on the above theoretical stance, we selected 12 key national policy documents released between 2008 and 2015 in England and 2007–2019 in Japan (Table 2). The policy documents were selected to allow for insights into the key developments of EOLC policies since the first publication of major EOLC policies in both countries, namely the *2008 End of Life Care Strategy* in England and the *2007 Guidelines on the Decision-making Process for Medical Care at End of Life* in Japan. These documents provided rich data for us to critically examine both the ongoing policy construction and the existing policy gaps in supporting of PCA in EOLC in both countries. A similar data collection approach was adopted to select five key legislations released between 2000 and 2017 in both countries (Table 3**)**, to illustrate the fundamental (and sometimes changing) judicial stance on individual rights and obligations regarding the care for dying patients and their support networks. Given the extensive number of policies and guidelines available at national, regional and organisational levels (especially in England), some documents that were referred for obtaining background knowledge in our study have not been included in Tables 2 and 3.

**Table 2:** Key EOLC policy documents

Policy	Country & year	Policymaker	Link
The Life Care Fifth Report of Session 2014–15	**England, 2015**	**House of Commons**	https://publications.parliament.uk/pa/cm201415/cmselect/cmhealth/805/805.pdf
One Chance to Get It Right: Improving people’s experience of care in the last few days and hours of life	**England, 2014**	**Leadership Alliance for the Care of Dying People**	https://assets.publishing.service.gov.uk/government/uploads/system/uploads/attachment_data/file/323188/One_chance_to_get_it_right.pdf
Report of the Mid Staffordshire NHS Foundation Trust public inquiry (Francis Report)	**England, 2015**	**House of Commons**	https://www.gov.uk/government/publications/report-of-the-mid-staffordshire-nhs-foundation-trust-public-inquiry
Policy for Prosecutors in Respect of Cases of Encouraging or Assisting Suicide	**England, 2010**	**Crown Prosecution Service**	https://www.cps.gov.uk/legal-guidance/suicide-policy-prosecutors-respect-cases-encouraging-or-assisting-suicide
Living Well with Dementia: A National Dementia Strategy	**England, 2009**	**Department of Health and Social Care (DHSC)**	https://assets.publishing.service.gov.uk/government/uploads/system/uploads/attachment_data/file/168220/dh_094051.pdf
End of Life Care Strategy	**England, 2008**	**DHSC**	https://assets.publishing.service.gov.uk/government/uploads/system/uploads/attachment_data/file/136431/End_of_life_strategy.pdf
Dementia Policy Promotion Charter.	**Japan, 2019**	**Ministerial Conference on the Dementia Policy Promotion**	https://www.kantei.go.jp/jp/singi/ninchisho_kaigi/pdf/shisaku_taikou.pdf (in Japanese)
Report on the survey of attitude toward medical and nursing care in the last stage of life.	**Japan, 2018**	**Ministry of Health, Labour and Welfare (MHLW)**	https://www.mhlw.go.jp/toukei/list/dl/saisyuiryo_a_h29.pdf (in Japanese)
Report on the public awareness of medical and nursing care at the Last Stage of Life.	**Japan, 2018**	**MHLW**	https://www.mhlw.go.jp/file/05-Shingikai-10801000-Iseikyoku-Soumuka/0000200748.pdf (in Japanese)
Basic Plan to Promote Cancer Control Programmes.	**Japan, 2007 (last revised in 2018)**	**MHLW**	https://www.mhlw.go.jp/file/06-Seisakujouhou-10900000-Kenkoukyoku/0000196975.pdf (in Japanese)
The Guideline on the Decision-making Process for Medical and Nursing Care at the Last Stage of Life.	**Japan, 2007 (last revised in 2018)**	**MHLW**	https://www.mhlw.go.jp/file/04-Houdouhappyou-10802000-Iseikyoku-Shidouka/0000197701.pdf (in Japanese)
Commentary: The Guideline on the Decision-making Process for Medical and Nursing Care at the Last Stage of Life.	**Japan, 2007 (last revised in 2018)**	**MHLW**	https://www.mhlw.go.jp/file/04-Houdouhappyou-10802000-Iseikyoku-Shidouka/0000197702.pdf (in Japanese)

**Table 3:** Key legislations

Legislation	Country & year of enforcement	Lawmaker	Link
The Care Act.	**England, 2014**	**UK Parliament**	https://www.legislation.gov.uk/ukpga/2014/23/contents/enacted
Health and Social Care Act.	**England, 2012**	**UK Parliament**	https://www.legislation.gov.uk/ukpga/2012/7/contents/enacted
Mental Capacity Act.	**England, 2005**	**UK Parliament**	https://www.legislation.gov.uk/ukpga/2005/9/contents
Cancer Control Act.	**Japan, 2007 (last revised in 2016)**	**National Diet**	https://elaws.e-gov.go.jp/document?lawid=418AC1000000098_20161216_428AC0000000107 (in Japanese)
Long-Term Care Insurance Act.	**Japan, 2000 (revised 2017)**	**National Diet**	https://elaws.e-gov.go.jp/document?lawid=409AC0000000123 (in Japanese)

(All documents in Tables 2 and 3 were accessed on 30th October 2021.)

### Data analysis

**Fig. 1:**
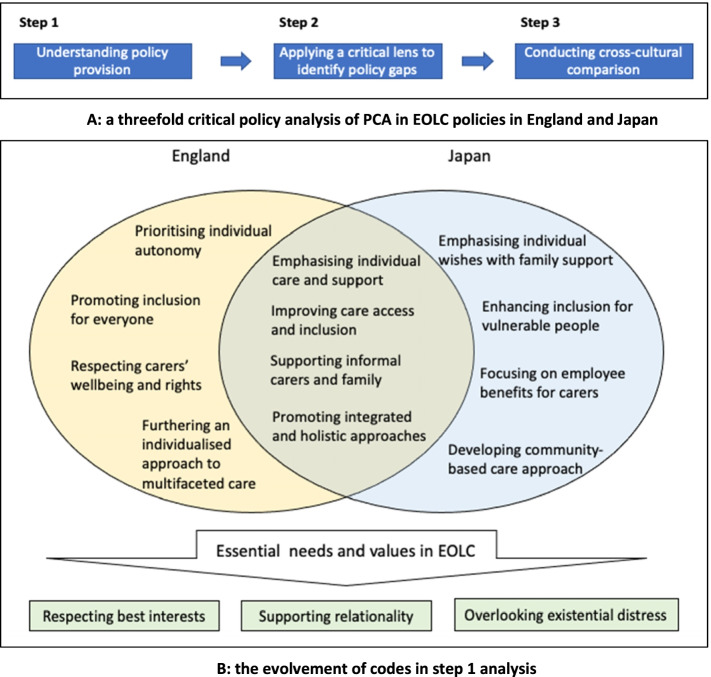
**A** threefold critical policy analysis of PCA in EOLC policies in England and Japan. **B**. The evolvement of codes in step 1 analysis

We critically analysed the above key EOLC policy and legislation documents to identify gaps between policy provision and needs/issues in reality following the process as outlined in Fig. 1**A**. In *step 1*, both authors independently coded policy documents respectively from England and Japan to analyse the meanings of PCA and the prescribed support structures. As illustrated in Fig. 1**B,** a series of initial codes were identified to capture both the shared and distinct policy provision for person-centred EOLC needs in both countries. These codes were then compared, refined and subsequently categorised into more encompassing themes to capture the multifaceted nature of PCA in EOLC policies. These three overarching themes could also allow for smoother comparisons of findings across the two cultural settings.

We subsequently moved to *step 2*, in which we developed a critical lens to examine PCA in EOLC, exploring the difference between the unique policy rhetoric (as macro-level structures) and practical realities (as micro-level investigations of people’s perceptions and experiences in healthcare, social care, legal and societal settings) [25]. By referring to the existing evidence reported in varied outlets (e.g., empirical studies, policy evaluations, literature reviews and grey literature) and examining this in relation to our three key themes, we identified gaps regarding how policies have (or have not) adequately addressed the complex and often interconnected needs and values in EOLC. We further compared these policy gaps in *step 3* by contextualising our findings (including policy provision and gaps) in the unique socio-cultural contexts of England and Japan, to clarify both shared and unique challenges/opportunities facing both countries.

To ensure the smooth progression of the above analysis activities, we (CF and MT) met frequently throughout the analysis process to discuss and compare findings at each of the above steps. If there was disagreement or divergence of analysis findings, further reading and discussion were conducted until a consensus was reached (both authors are fluent in English and Japanese). As such, we developed a cooperative and reflexive approach to contest and further enrich what it means to be a person in EOLC settings of both individualistic and collective societies, contributing to the ongoing (re) constructions of PCA in EOLC policies and practices.

### Findings

We found that the term ‘person-centred approach/care’ was relatively absent in the selected English and Japanese policy documents. Rather, it was often conceptualised as a vague goal for EOLC delivery. Three primary themes were identified: ‘respecting best interests,’ ‘supporting relationality,’ and ‘overlooking existential distress.’ These three themes illustrated that the EOLC policies in both countries do not only focus on individual and interpersonal/societal dimensions of PCA but also pay increasing (but inadequate) attention to the deeper (more existential) needs in EOLC. Taking a critical approach, we drew upon evidence from academia and wider society to further question these policy constructs of PCA within the complex structural and individual circumstances of EOLC (where the policies are made and implemented). As such, we uncovered how socio-political, cultural, organisational and individual contexts may shape the ongoing (re) constructions of EOLC policies, highlighting policy gaps in recognising and supporting complex person-centred needs in EOLC.

### Respecting best interests

How to prioritise patients’ individual interests lies at the heart of EOLC policies both in England and Japan. These individual needs and values are often interpreted as ‘autonomy’, ‘choice’, ‘welfare’ and ‘dignity’ [15]. While policy provision varies in both countries, the aim to empower patients’ individual agency in EOLC and also to ensure their wishes are effectively addressed when incapacitated (e.g., loss of mental capacity) remains key. We also found that how policies construct individuals’ best interests and the subsequent means of supporting these interests also strongly conform to broader socio-cultural values. Given the largely subjective nature of best interests however, policies in both countries are faced with ambiguous interpretations of patients’ individual needs and preferences. These ambiguities are further compounded by the unique English and Japanese cultural values which often lay different emphases on individuality, giving rise to distinct policy gaps in addressing best interests in both contexts.

#### England

A core principle throughout the English EOLC policy documents is to ensure the best interests of dying patients [19]. The *End-of-life Care Strategy* (hereafter the *Strategy*) sets out a comprehensive framework to ‘have their [patients’] needs assessed, their wishes and preferences discussed, and an agreed set of actions reflecting the choices they make about their care recorded in a care plan’ (p.12). This emphasis is consistently reflected in the following policies. The *Francis Report* calls for a ‘patient-first’ healthcare culture within all healthcare organisations in response to the Mid Staffordshire NHS Foundation Trust’s structural failures to protect patients’ interests. In addition, the *Fifth Report* urges the government to tackle the shortfall of district nurses and outreach care facilities in communities to help people die at their preferred place in the company of their loved ones (p.30).

Formalised mechanisms have also been developed in England to ensure dying patients’ best interests are communicated and respected in diverse circumstances. ACP, as a key means, has been consistently advocated in EOLC policies, encouraging people to discuss and record their preferences for EOLC with family and practitioners before losing the decision-making capacity. Care planning is particularly emphasised for dementia patients with early onset of their illness by the *EOLC Strategy*, the *Living Well with Dementia Strategy* and in other documents. Central in the ACP discourse is to maximise patients’ chance of receiving care that reflects their best interests [23]. Although ACP documents are not legally binding, as addressed in the *Fifth Report* and other policies included in this study, people’s care plans ‘must be taken into consideration’ (p.24). This policy stance is further reinforced by *the Mental Capacity Act*, which provides legal warrants for people’s right to refuse particular treatments and to appoint a lasting power of attorney to make decisions on their behalf.

Despite being extensively emphasised, the boundary of best interests as a subjective perception remains largely blurred in policies. For example, euthanasia and assisted dying are prohibited in England, although travelling abroad for assisted dying is not illegal [26]. The *Policy for Prosecutors* provides a strict warning and detailed advice to prevent physicians and families from assisting patients to end their lives. Opposition has argued that patients should be able to terminate their own life as a basic right to maintain dignity and agency [26]. The ongoing debates on the right to die have highlighted the difficulties facing policymakers and legal structures in adequately defining and supporting patients’ best interests from their own perspectives [27].

This challenge is also captured by the discontinuation of the Liverpool Care Pathway (LCP). As a clinical pathway designed to pursue a patient’s best interests in the last days or hours of life, LCP was widely endorsed by the *Strategy* and following policies. However, it was suddenly phased out in 2014, following a national independent review in response to increased criticism. The criticism largely focused on LCP’s standardised approach (e.g., ticking boxes) and clinicians’ authority over deciding terminal sedation of patients, arguing this may violate patients’ rights [28]. The Leadership Alliance for the Care of Dying People was formed shortly after, and the *One Chance to Get It Right* report was released, proposing five priorities for promotion of a more individualised approach to assessment and care of patients nearing death. Despite continued exploration of clinical pathways for dying patients, this dispute about LCP has highlighted the ambivalent understanding of best interests in English EOLC policies.

Policies have also inadequately addressed structural barriers to access sufficient care to pursue their best interests. Despite the ongoing policy commitments to eliminate inequalities in EOLC, such as the *Strategy* and the *Fifth report*, it is found that many patients are admitted to palliative and hospice care units only shortly before death due to lack of timely and readily available support for patients with early-stage diagnosis [29]. Social inequalities and unevenly distributed medical resources are also contributors that are persistently deterring EOLC access for those from deprived backgrounds, ethnic minorities and non-cancer patients [8]. Structural barriers are also evident in the means of communicating the best interests. For example, regardless of the strong policy advocacy and increasing uptake of ACP, as reported in 2020, less than 5% (4.8%) of people admitted to emergency care in the UK had ACP, this largely mirrors a national picture that only 5% of the general population have an ACP or formal living will [30]. These barriers are also evident in the problems seen with interoperability and data sharing between care providers [31].

#### Japan

Compared to England, Japan has released relatively limited policies that focus on dying patients’ best interests. The most explicit explanation of individual interests in EOLC is found in *the Guideline for Medical Decision-making Process* (hereafter, the *Guideline*)*,* which was initially released in 2007. As the first EOLC policy**,** *the Guideline* explicates that respecting patients’ self-determination should lie at the heart of the principles and mechanisms to inform medical care at the end of life (p.2). In addition, the roles of healthcare professionals and family members are also frequently highlighted in the *Guideline.* Hence, deciding what is best for patients is largely undertaken as a joint endeavour often involving important others. This collective nature of decision-making, particularly the role of family, becomes more evident when patients lose the capacity to express their wishes. A similar approach to advocating patients ‘best interests’ can also be observed in the *Dementia Policy Promotion Charter*, stipulating that dementia patients’ best interests (including prevention and continuous support) should acknowledge the needs and views of both patients themselves and their family members (or informal carers).

Despite the above policy instructions on evaluating and supporting patients’ best interests, legislation to ensure patients’ autonomy in decision-making remains largely absent in Japan. While active euthanasia and assisted suicide are illegal in Japan, there are no legal instructions and mechanisms to guarantee patients’ rights and dignity and to guide healthcare professionals. As a result, certain requests (e.g., forgoing life-sustaining treatments) may cause difficulties and controversies pertaining to EOLC. A well-known case was the 1995 Tokai University Hospital Case, where an attending physician withdrew treatment from a 58-year-old patient suffering from end-stage multiple myeloma at the Tokai University Hospital. The physician then administered sedative drugs to stop the patient from stertorous breathing, followed by potassium chloride, which causes heart failure if injected undiluted. The patient subsequently died from cardiac arrest, and the attending physician was prosecuted for homicide. The district court convicted the attending physician with a suspended sentence. In the ruling, the Court not only set forth admissibility requirements for active euthanasia, but also provided admissibility criteria for forgoing life-sustaining treatments in the *obiter dictum* [32]. This case attracted wide attention contesting the legal and ethical boundary of patients’ autonomy in EOLC; however, this ambiguity persists in current legal systems in Japan.

Besides lack of legal guidance on withdrawal of treatments and for preventing further controversies, some legal scholars have argued that specialised palliative care, including palliative sedation, can alleviate patients’ pain at the end of life, and thus, may minimise their intention to request the forging of life-sustaining treatments or euthanasia [33]. The *Guideline* document calls for a rigorous medical approach to ensure that ‘the medical and nursing care team should carefully decide the medical validity and ethical propriety of starting or not starting, changing or withdrawing any specific medical or nursing care’ (p.3). Further, to reduce the predominant role of healthcare professionals in the above decision-making process, the *Commentary* for the *Guideline* (hereafter the *Commentary*) was revised in 2018 to include ACP as a potential solution to better support patients’ wishes and dignity (p.1).

While acknowledging the emerging policy emphasis on patients’ best interests, lack of broader social discourses to recognise and further support patients’ autonomy in Japanese society is the core and current challenge. According to the *Report on the Survey of Attitude towards Medical Care in the Last Stage of Life*, 40–70% of respondents had no knowledge of ACP. It also revealed that 30–50% had not yet had conversations about their preferences for end-of-life medical care with family members or healthcare providers. Another survey [34] revealed that only 25% had opportunities to talk about EOLC arrangements with their loved ones, although 66% of respondents wished to do so.

### Supporting relationality

English and Japanese EOLC policies also expand the parameters of PCA beyond the individual paradigms of patients’ best interests. They do so by integrating both patients’ relational needs and the multifaceted needs of their close others into the principles and support mechanisms in varied EOLC settings. Central to this relational policy approach is to clarify that EOLC is not an individual matter but involves various parties in the patients’ family and wider social networks [19]. We found a variety of policy guidance and measures that have developed in the two countries, to ensure EOLC as a joint experience in which both patients and their close others need to be supported. Despite this inclusive scope, the policy objectives to support relationality in EOLC are often obscured by the complexity of coordinating varied and even competing needs and preferences from patients and others, especially within limited resources. From a cultural perspective, the different social scripts on the role of family and (in)dependence also prompt unique challenges for policy discourses in both countries to define and address relationality in EOLC.

#### England

In addition to the individual’s best interests, English policies seek to address EOLC needs in the complex matrix of the patient’s family and social networks [35]. Starting from the *Strategy*, policies have consistently acknowledged the vital roles of ‘[t]he family, including children, close friends and informal caregivers’ (p.14). Family involvement is believed to allow patients to be accompanied by close others which helps the patients achieve a ‘good death’ [23]. Support for patients’ relational needs is also reflected in the emphasis on family involvement in EOLC decision-making processes. In light of the controversies caused by LCP, the *One-Chance Report* prioritises involvement of the family members in communicating about and making decisions about the patient’s care throughout the dying process. The *Mental Capacity Act* also provides legal mechanisms for the lasting power of attorney to ensure that patients’ wishes are communicated by trusted family members or others when themselves are not in a position to do so. Beyond healthcare, the *Fifth Report* urges for additional governmental inputs into social care provision, including improvement of community-based palliative care resources and provision of free social care packages to meet patients’ relational need to be with their family and in their home environment.

Policy discourses also tend to view EOLC needs as compounded by both patients and their close others. Apart from the *Francis Report*, all other policies included in this study strongly emphasise the importance of supporting the welfare of families and the informal/unpaid carers, such as provision of individualised support and respite care. The *Care Act* defines carers’ legal rights to access support, clarifies local authorities’ responsibilities to assess carers’ diverse needs and subsequently provide suitable support. At the national level, Carer’s Allowance is provided to assist low-income carers. The continuation of support for families and caregivers even after the patient’s death can also be found in the current policy discourses, including bereavement care through care providers and the non-means-tested Bereavement Support Payment. The *Strategy* emphasises on the need for care facilities to provide resources and workforce training to ensure comprehensive and socio-culturally appropriate bereavement care. The *Fifth Report* also points out that further structural and funding improvements are required for the improvement of the ‘very fragmented and inequitable’ bereavement support across the country (p.36).

Various barriers have been identified in both health and social care settings, contradicting the ethics of relationality embedded in the above policies. Fundamental in these relational barriers is the predominant social emphasis on autonomy, which may subsequently lead to underestimating patients’ and their families’ needs for (inter)dependency [19]. Such an emphasis can also create barriers in inadequately communicating patients’ and their families’ needs for external professional support [36]. This miscommunication pertaining to independence can be more prevalent among ethnic minorities when practitioners misinterpret their collective socio-cultural values and customs, leaving their relational beings less supported [8]. The patients’ need to be accompanied by loved ones in their familiar surroundings can also be challenged by the discontinuation of care (e.g., inconsistent access to services across organisations). Such complex support structures can further overstretch community palliative care resources, thus preventing patients from receiving home and community-based care and subsequently draining the hospital resources [5].

One of the most prominent challenges to the relational needs of EOLC is the lack of affordable social care. These support gaps resonate within the neo-liberal policy agendas, encouraging patients and caregivers to be self-sufficient in arranging care and thus reducing welfare dependency [19]. Currently, patients can only access government-funded/subsidised social care services if they are economically vulnerable and have passed through prolonged assessments [37]. As such, an increasing number of patients and their families are forced to exhaust savings to fully or at least partially cover their long-term social care costs [37]. This insufficient state provision of care is likely to cause growing pressure on patients and their families/carers, strongly limiting their capacities to focus on their relational needs. Furthermore, it can be particularly burdensome for the informal carers with reduced capacities, such as older caregivers (spouses) of dementia patients, but their concerns are yet to be addressed [38].

#### Japan

The major Japanese EOLC policies also entail a relational dimension addressing PCA in the complex relationships involved in EOLC. The *Guideline* briefly emphasises that dying patients’ needs are not only medical but also ‘social’ (p.2). The *Commentary* highlights the need of social work and other multifaceted supports to meet the patients’ social needs. These policies also emphasise relational needs by recognising the need to enable patients to obtain support from trusted others (family and healthcare professionals) at difficult times. For example, the *Guideline* stipulates that the medical validity and ethical propriety of EOLC treatments should be determined through repeated discussions among the medical care team, family members and the patient if possible. Moreover, the emphasis on the family agency is particularly strong as the *Guideline* permits trusted family members to presume the patient’s best interests when the patient has lost their mental capacity (p.3). Considering the increase in single households, the definition of ‘family’ to include friends allowing multiple trusted people to support the patient in the EOLC process has been expanded in the *Commentary* (p.6).

Another aspect of the relational needs acknowledged by Japanese policies is the support for families and the informal carers. Both the *Guideline* and the *Commentary* provide overarching principles to ensure appropriate support structures for family members and close others during EOLC. Regarding social care, Japan has developed the Long-Term Care Insurance (LTCI) to provide need-based support for people aged over-65 s and for patients aged 40–64, who need intensive care because of age-related or terminal diseases. The eligible patients can access different levels of support (e.g., short-stay and day-care services, home-based care). Based on ‘the principle of the cooperation of citizens’ solidarity’ (LTCI Act, Article 1), this non-mean-tested LTCI aims to use collective resources in relaxing the caregiving burden of family members and informal carers. In addition, a series of legal guarantees, such as the Employment Insurance Act and the Child Care and Family Care Leave Act ensure that employed carers get access to paid leave.

The complex needs and relationships of EOLC, however, need to be further addressed. In health care, the strong emphasis on EOLC as a joint endeavour between the patient, family members and practitioners may cause disruptions in planning, decision-making, and subsequently, the quality of care. ‘Relational autonomy’, which emphasises individual autonomy in the surroundings, has been widely seen in EOLC settings [39]. Given the traditional family-centred values and the authoritativeness of medical professionals, it was found that patients may feel reluctant to challenge shared decisions and express their own wishes [40]. As such, patients are likely to face cultural barriers while addressing their needs in a truly cooperative manner [39]. Even in well-constructed social care systems, the needs of patients and their families cannot always be met. For example, despite providing universal coverage, LTCI does not cover patients under 40 or those over 40 having care needs due to acute conditions [37]. Respite and bereavement care are also not included in the LTCI and other care systems. According to a survey of bereaved families [41], nearly half of the respondents felt the burden of care heavily, and about a third felt prolonged bereavement due to lack of sufficient social care services.

### Overlooking existential distress

Policies from England and Japan pay further attention to deeper dimensions of person-centred needs in EOLC. These needs may not only be faith-related or derived from specific emotional/social conditions, but also involve more fundamental deprivation of meaning as they approach the end of life [20]. That is, one might encounter existential distress (ED), a deeply painful fear that one’s lives, memories, narratives and identity are increasingly forgotten, unimportant, unvalued and irretrievably lost in the past [42]. As pointed out by Bolmsjö et al. [43], ‘the patient’s existential needs is [are] experienced as one of the greatest challenges for healthcare personnel’ (p.1311). While acknowledging the significance of supporting patients’ needs beyond the paradigms of specific individual meanings and inter-personal relations, the EOLC policies in both countries, as we found, tend to overlook the complexities about existential distress in EOLC. Often inadequately categorising these complex deeper needs into ‘religious needs or psycho-social needs, the current policies are likely to prevent the two countries from achieving PCA more deeply and holistically in EOLC.

#### England

The English policy documents primarily used the word ‘spiritual’ to underline two realms of deeper needs: religious and existential [20]. While emphasising the importance of religious needs of patients and their family, the *Strategy* advocates, ‘[t] hey should be able to express their hopes and expectations of what has deepest meaning for them’ (p.76). It further uses the Hereford St Michael’s Hospice as an example to briefly showcase how spiritual needs related to ‘regret, meaning, value, and purpose’ can be assessed and supported (p.77). However, no further explanations about patients’ existential needs are provided in the *Strategy* and other policy documents included in this study. Consequently, the policy construct of spiritual care, as suggested by the *One-Chance Report*, is predominantly ‘culturally and religiously specific’ without concerning the patients’ complex and deeper pain of meaninglessness and alienation (p.90).

The inadequate policy interpretation of existential needs also lies in the specialisation of spiritual care. As addressed in the *Strategy*, the *One-Chance Report* and the *Fifth Report*, spiritual support tends to be narrowly defined as ‘specialised care’. Similar to psychological and medical care, spiritual care often involves formal assessments and interventions rather than being provided as part of daily care. This specialisation is also evident in the policy emphasis made on chaplains/chaplaincy. The *One-Chance Report* highlights chaplaincy departments’ roles in conducting staff training for spiritual care and in coordinating care provisions to meet the cultural and religious needs (p.33, 90). While acknowledging the significant contributions of chaplains and chaplaincy, little emphasis has been placed on the roles of other care practitioners and family members in reinforcing spiritual resilience.

The overlooking of ED in policy discourse reflects and further exacerbates the lack of ongoing structures to address patients’ existential needs in English EOLC provisions. To date little English/British EOLC research has particularly focused on ED, while studies from other western (mostly Northern European) countries have addressed various common challenges for ED. These studies have suggested that the lack of support for patients facing ED is a compounded issue. First, little guidance is available for practitioners to understand patients’ ED [44]. Second, the emphasis on autonomy in EOLC may serve as a cultural barrier impeding practitioners’ motivation to address the highly personal ED experiences of their patients, while patients may also feel reluctant to disclose their deeper pains [20]. Third, practitioners’ busy workloads may also reduce their opportunities to better understand their patients’ lives and deeper concerns about indifference and abandonment [43]. Finally, the highly professionalised structure of EOLC may also underestimate the role of patients’ significant others (e.g., spouse, children), thus failing to incorporate their insightful views about the patients’ lives into care [42]. Hence, it can be suggested that ED has been inadequately envisaged in EOLC in England as compared to Netherlands, where decisions on continuous deep sedation in EOLC can be taken on the basis of the extent of existential suffering of the patients [45].

The current lack of comprehensive workforce education regarding ED in EOLC practice is another key challenge in England. Practitioners who are most in touch with patients (e.g., doctors, nurses) are often not educationally prepared to recognise and support the patients’ existential needs [20]. This raises questions as to how to further equip practitioners (both within and outside EOLC) with compassionate skills and tools to accompany patients through their suffering [46]. However, significant structural barriers persist, such as workforce/funding shortfalls and issues regarding inter-organisational cooperation. These issues can inevitably restrict practitioners’ abilities to learn and deliver consistent and coordinated support for the patients’ existential needs.

#### Japan

There is lack of emphasis on the necessity of dealing with ED in EOLC in Japanese policies. The *Guideline* only stipulates that ‘the healthcare team must provide comprehensive medical and nursing care … and offer psychological and social support to the patient and his/her family’ (p.1). The *Commentary* simply states that ‘when a person approaches the end-of-life stage, other kinds of psychological and social problems may be accompanied as well as pain relief. If possible, the healthcare team should involve a social worker or another person competent in dealing with the social care aspects, or a care assistant’ (p.4). As such, these two policy documents do not explicitly address spiritual or existential needs while administering EOLC, but broadly categorise patients’ deeper pains of meaninglessness and impending death as socio-psychological needs.

Existential needs have long been acknowledged in clinical settings. Research focusing on Japan reveals that psycho-existential suffering can serve as a crucial indicator of the need for palliative sedation therapy [47]. In a broader context, research also highlights the importance of developing mechanisms that support dying patients to live well by alleviating varied psychological suffering, such as feelings of meaninglessness, purposelessness, loneliness, anxiety, alienation and dependence [48]. It has also been argued that Japanese physicians’ intentions to provide palliative sedation not only focus on patients’ physical suffering, but also on their deeper distress to facilitate a ‘good death’ [47].

As mentioned above, ED may be attributed to a lack of genuine communication to express the complex and changing pain felt as one approaches death [43]. Murata [49] also points out that communication is key that spiritual care can alleviate the existential pains associated with ‘the disappearance of the self’ in this world and the ‘disconnection from others’. The Good Death Inventory (GDI) is another channel that can recognise ED in clinical settings. Initially developed in Japan, the GDI is a validated measure for evaluating the quality of care for terminally ill cancer patients from the perspective of close family members [50]. It concerns various and often interrelated factors that are essential for addressing the ED needs of patients and their loved ones, including ‘life completion,’ ‘dying in a favourite place,’ ‘maintaining hope and pleasure,’ ‘not being a burden,’ ‘good relationship,’ ‘being respected as an individual,’ ‘religious and spiritual comfort,’ ‘control over the future,’ ‘feeling that one’s life is worth living,’ ‘pride and beauty,’ ‘natural death,’ ‘preparation for death,’ and so on [50].

Therefore, it is evident that the need to identify and support ED has already received attention in clinical settings in Japan. However, government policies are yet to adopt both the empirical evidence of patients’ deep pain and the existing mechanisms (e.g., GDI) to propagate adequate support structures and educational resources for such complex and evolving needs.

## Discussion

Our analysis provided further insights into the holistic meaning of PCA within two socio-culturally distinctive contexts. While EOLC needs are more explicitly addressed within the paradigms of dying patients’ individual lives and social/support networks, their existential needs for more fundamental meaning in facing suffering and death were also captured. These findings reaffirmed and further extended the conceptual boundaries of PCA in EOLC policies in the following three dimensions.

### Individual dimension

We found that the key EOLC policies in England and Japan have largely mobilised PCA focussing on the individual, seeking to promote the goals and means of addressing patients’ individual needs, dignity and autonomy in various settings. As widely captured in both countries, extensive narratives for propagation of PCA by supporting the patients’ best interests of have been illustrated in the national guidelines/strategies and legislations. As a primary policy concern, the ‘best interests’ have not only been interpreted in various forms (e.g., preferences for care, self-determination), but have also been prescribed/promoted as different legal and practical mechanisms for ensuring patients’ individual being to be supported at different stages of dying.

Despite the available policy developments in both countries, the patients’ best interests can be inadequately understood by policies and easily interrupted by both external structures (e.g., bureaucratic barriers) and individual subjectivity (decision-making by others). Central to this policy dilemma is the difficulties in explicitly defining ‘best interests’, including who should determine the patients’ best interests and how to safeguard this process. For instance, the *Mental Capacity Act* in England does not specify the definition of best interests but rather provides various means ensure the patients’ needs and preferences to be considered. Similarly, the Japanese *Guideline* points out that the best option representing each patient’s interests should be chosen after careful discussion among patients, their families, and care professionals. In addition, the lack of consistent structures to encompass the complexity around the perspectives of patients, close others and healthcare professionals remains unresolved; this has been clearly evidenced by the low prevalence of ACP in both countries.

Due to the above ambiguity in the policy constructs, both countries face challenges as to who should have the final say regarding patients’ best interests. This challenge is compounded by the lack of well-coordinated structures to ensure the communication and transfer of information. Despite the similarities, the analysis also revealed that both countries were at different stages of policy development regarding the best interests. It seems that the English policy provision is more detailed and comprehensive, whereas that of Japan still need improvement, specifically to promote awareness of the importance of individual interests and autonomy among the public and the care professionals. Our analysis has also revealed cultural differences in understanding best interests, which are generally emphasised as an individual matter in England, while in Japan are often considered a joint task involving patients, families, and practitioners [23, 47]. The latter is commonly indicated in other East Asian cultures, focusing on family-centred decision-making [32]. This cultural disparity also reflects different cultural norms regarding the meaning of ‘individuals’, further highlighting the importance of a culture-competent approach to patients’ best interests (e.g., decision-making and advance planning).

Based on the above comparison, further policy developments are needed in both countries to enhance the ways of communicating best interests in EOLC. It is worth noting that we do not intend to criticise the lack of a unified definition of ‘best interests’, as the personal needs and values are often subjective and unique. Instead, we argue for an ongoing structure to adequately understand the complex needs of dying patients. Both countries need to improve clarity and make the current policies more flexible to eliminate the structural barriers in the care systems and to facilitate understanding and respect for the best interests. Japan can learn from England to further develop dedicated policies and formal structures, such as comprehensive national strategies and ACP programs, to accommodate the patients’ best interests in both EOLC and broader society. England may benefit from a more collaborative approach, as evidenced in Japan, to enable more negotiations among patients, families, and practitioners to avoid conflicts.

### Relational dimension

Our analysis also captured a profound relational dimension of PCA in EOLC policies. Both England and Japan have developed policy and legal mechanisms to emphasise the relational nature of the patients’ experiences in EOLC. Both countries have acknowledged the relational needs of the patients to ensure support from close others alongside their illness trajectories (e.g., decision-making supported by family, dying in a homely environment and community-based support). There are also systems in place to support the needs of close others (including family members and/or informal carers) both in England and Japan, helping them stay engaged and involved during the patient’s last stage and beyond. Despite the policy provision supporting relational needs in EOLC, policy gaps have been identified in both countries, showing significant discrepancies in social care provisions in England and lack of diversified support for close others in Japan.

Significant cultural differences have been captured in the policy constructs of relational needs in EOLC. In the English policies, despite the emphasis on supporting patients and their close others as relational beings, the cultural emphasis on individuality and independence may undermine the recognition for support and care from others [19]. This individualised focus in EOLC policies may not fully reflect the various needs of patients for relationality in ethically and culturally diverse English society [8]. Compared to the competing policy interests between individuality and relationality in England, Japanese policies show a stronger emphasis on ‘relational autonomy’ in EOLC, promoting a highly negotiated process of decision-making in consensus-building among the patient, family members, and physicians [39]. Such ‘relational autonomy’ may suppress the individualised values of patients in Japan, highlighting the complexity of providing adequate support to both individual and relational needs in EOLC.

Cultural differences are also closely embedded in English and Japanese policy agendas for relational needs in EOLC. In general, England has developed comprehensive policies to accommodate the relational needs of patients and to support their families and caregivers. However, as compared to Japan, the English policies (e.g., means-tested social care) resonate with a larger neo-liberal agenda, which conforms to self-reliance and further aims to reduce people’s welfare dependency [19]. As such, relationality in EOLC has been paradoxically interpreted by the English policies, and have failed to sufficiently incorporate both the dependent and independent aspects pertaining to the values and needs of the patients and their families. Conversely, policy support in Japan emphasises interdependence. This policy agenda is particularly evident in the universal LTCI, which is fundamentally based on the principle of cooperation between citizens and solidarity.

Considering the different cultural and policy approaches to supporting relationality in EOLC, England and Japan face distinctive challenges in reducing their policy gaps. English policymakers should learn from the Japanese to explore the potential for establishing a more universal social care system within the English context (rather than simply replicating the Japanese system) [37]. A social care system that is not mean-tested may enable patients and their loved ones to spend quality time in their preferred environment, without experiencing intensified financial pressure and undergoing prolonged bureaucratic procedures. Another aspect of policy improvement in England is the development of a more culturally competent and flexible policy structure to support patients and their families from different backgrounds (e.g., ethnic minorities and immigrants). The primary challenge facing Japan is fragmented policy support for relationality, which urgently requires more multifaceted care for both patients and their families (e.g., respite care and bereavement support). The policies in Japan should aim for meeting the increase in multicultural demands for EOLC amid its increasingly diversified population (e.g., immigrants). Despite the differential policy gaps, it is pivotal to balance relational and individual needs in EOLC, not as separate issues but as interrelated entities encompassing people’s values and life histories.

### Existential dimension

Beyond the individual and relational aspects, we identified an existential dimension of PCA in EOLC policies. While policies in both countries have recognised spiritual and/or other deeper socio-psychological needs, the conceptual understanding and practical support mechanisms for ED are inadequate and fragmented. This policy gap is evident in the observations made in clinical settings (e.g., experiencing deep pain of losing meaning and purpose in life, and feeling indifferent and forgotten alongside dying) [20, 43]. Whilst little evidence is directly collected from England, due to its deeply painful nature, ED is seemingly a common experience among patients receiving EOLC. Such experiences may be further amplified by the highly institutionalised healthcare systems and the cultural emphasis on autonomy in the western context, which can undermine patients’ access and motivation to fulfil their existential needs [20]. A similar gap is also captured in Japan, further demonstrating the prevalence of existential pain among terminally ill patients and the significance of providing appropriate support (e.g. palliative sedation, the GDI) [47, 49, 50].

This policy gap is unique because England and Japan are both faced with the deprivation of adequate policy coverage for ED. Fundamental to this shared policy gap is the highly abstract nature of such deeper pain that can be difficult to communicate and be adequately supported [42, 43]. Despite this, both countries have already developed policy discourses to ensure holistic care and interpersonal communication as crucial bases for improving awareness of ED in EOLC settings and wider society (e.g., chaplaincy-based care and psycho-social support related to non-medical needs). Education is key to future policy developments in both countries to proliferate the literacy of existential needs among practitioners, patients, families and the public. A more systematic and ongoing structure is also needed to coordinate the support for ED in EOLC (e.g., platforms for patients to share their concerns and fears, inter-organisational cooperation and data-sharing).

In addition to these shared policy improvements, culturally competent policies are equally crucial for addressing ED that is often experienced and dealt within socio-cultural processes [51]. Thus, policies supporting ED should reflect the cultural values to shape patients’ fundamental sense as a ‘person’, enabling them to feel valued, remembered and tenderly attended in EOLC and their everyday lives. In England where a ‘person’ is profoundly defined as an autonomous being, the policy developments should seek to balance the emphasis on respecting patients’ independence and providing compassionate and culturally-competent support for addressing their existential needs. In the Japanese context, patients’ existential needs should be accommodated in alignment with the predominant cultural identity conforming to family and collective values, enabling them to feel interdependent and supported. Another avenue for Japanese EOLC policies is to explore how the prevalent cultural systems of spirituality and *ikigai* (a sense of purpose and fulfilment in life) may alleviate patients’ ED. To start developing ED-supportive EOLC policies, research from both quantitative and qualitative perspectives is urgently needed in England and Japan to provide sound evidence to policymakers.

### Limitations and implications

We acknowledge that our study is limited in the sense that, despite adopting an international perspective, we did not intend to carry out an exhaustive review of EOLC policies and the literature. Instead, we intended to use limited space to encourage critiques and reflections between EOLC policies and practice to propagate a more PCA in EOLC. Future research can explore beyond national policies to critically examine EOLC policies and their deployment of a PCA across more localised contexts (e.g., guidelines and programmes in varied clinical, occupational and organisational settings). We also believe our study (both the findings and the method) will be useful to inform studies that focus on EOLC policies in and/or across other socio-cultural contexts, to enrich the understandings and implementation of PCA in EOLC in a more socio-culturally competent manner.

## Conclusion

Based on the above analysis, we argue that the policy constructs of PCA should be extended to a whole-person approach not only in England and Japan but also more broadly. Most simply, PCA aims to prioritise the ‘person’. As such, policies should continue to focus on the individual being in EOLC by seeking to adequately prioritise patients’ complex interests to support their agency and dignity alongside dying. It is equally important to view patients as relational beings in policy discourses, as patients’ needs and values can shape and be shaped by their rich matrix of relationships and socio-cultural backgrounds. In addition to individual and relational beings, EOLC policies should begin to look deeper into patients’ existential being, as this is deeply embedded in their life histories. As one deteriorates and approaches the end of life, patients may feel forgotten, unimportant and inextricably lost; thus, policy objectives need to better acknowledge and support their deeper needs to help them retain their meaning and identity in a more fundamental sense.

As Cicely Saunders understood ‘total pain’ at the end of life, ‘a cry just to be rid of pain is not worthy of man … Man by his very nature finds that he has to question the pain he endures and seek meaning in it’ [17]. Thus, it is crucial that while developing policies in future, the holistic meaning of person-centredness in shaping EOLC should be integrated. For that, we believe both theoretical and empirical research is needed to (re) examine the contexts, texts and consequences of this extended whole-PCA in the EOLC policies.

## Abbreviations

ACP: Advance care planning
ED: Existential distress
EOLC: end of life care
GDI: Good Death Inventory
LCP: Liverpool Care Pathway
LTCI: Long-Term Care Insurance
PCA: Person-centred approach

## References

[1] Slater L (2006). Person-centredness: a concept analysis. Contemp Nurse.

[2] Nolan M, Davies S, Brown J (2004). Beyond person-centred care: a new vision for gerontological nursing. J Clin Nurs.

[3] Thomas K, Gray M (2018). Population-based, person-centred end-of-life care: time for a rethink. Br J Gen Pract.

[4] De Lima L, Pastrana T (2016). Opportunities for palliative Care in Public Health. Annu Rev Public Health.

[5] Clelland D, Steijn D, Whitelaw S (2020). Palliative Care in Public Policy: results from a global survey. Palliat Med Rep.

[6] Öhlén J, Reimer-Kirkham S, Astle B (2017). Person-centred care dialectics-inquired in the context of palliative care. Nurs Philos.

[7] Clark D, Takenouchi H (2020). The Mitori project: end of life care in the United Kingdom and Japan – intersections in culture, practice and policy. Progress in Palliative Care.

[8] Dixon J, King D, Matosevic T, et al. Equity in the provision of palliative care in the UK: review of evidence. Discussion Papers. London, UK: London School of Economics and Poltical Science; 2015. No:2894.

[9] Hirayama Y, Otani T, Matsushima M (2017). Japanese citizens' attitude toward end-of-life care and advance directives: a qualitative study for members of medical cooperatives. J Gen Fam Med.

[10] van Belle H (2005). Philosophical roots of person-centered therapy in the history of Western thought. The Person-Centered Journal.

[11] Entwistle V, Watt I (2013). Treating patients as persons: a capabilities approach to support delivery of person-centered care. Am J Bioeth.

[12] Pringle J, Johnston B, Buchanan D (2015). Dignity and patient-centred care for people with palliative care needs in the acute hospital setting: a systematic review. Palliat Med.

[13] Beauchamp T, Childress J (2019). Principles of biomedical ethics.

[14] Graven V, Petersen A, Timm H (2020). Hospice care: between existential and medical Hope. Mortality..

[15] Wilson F, Ingleton C, Gott M, Gardiner C (2014). Autonomy and choice in palliative care: time for a new model?. J Adv Nurs.

[16] Radbruch L, Leget C (2016). Euthanasia and physician-assisted suicide: a white paper from the European Association for Palliative Care. Palliat Med.

[17] Saunders C (1966). The care of the dying. Guy’s Hospital Gazette.

[18] Jeong S, Higgins I, McMillan M (2009). The essentials of advance care planning for end-of-life care for older people. J Clin Nurs.

[19] Borgstrom E, Walter T (2015). Choice and compassion at the end of life: a critical analysis of recent English policy discourse. Soc Sci Med.

[20] Ettema E, Derksen L, van Leeuwen E (2010). Existential loneliness and end-of-life care: a systematic review. Theor Med Bioeth.

[21] Tokuda Y, Nakazato N, Tamaki K (2004). Evaluation of end of life care in cancer patients at a teaching hospital in Japan. J Med Ethics.

[22] Torres S, Ågård P, Milberg A (2016). The ‘other’ in end-of-life care: providers’ understandings of patients with migrant backgrounds. J Intercult Stud.

[23] Borgstrom E. What is a good death? A critical discourse policy analysis. BMJ Support Palliat Care 2020;bmjspcare-2019-002173. 10.1136/bmjspcare-2019-002173 32631959

[24] Taylor S (1997). Critical policy analysis: exploring contexts, texts and consequences, discourse. Studies in the Cultural Politics of Education.

[25] Young MD, Diem S, Lochmiller C (2018). Doing critical policy analysis in education research: an emerging paradigm. Complementary research methods for educational leadership and policy studies.

[26] Richards N (2017). Assisted suicide as a remedy for suffering? The end-of-life preferences of British “suicide tourists”. Med Anthropol.

[27] Huxtable R (2014). Autonomy, best interests and the public interest: treatment, non-treatment and the values of medical law. Med Law Rev.

[28] Seymour J, Clark D (2018). The Liverpool care pathway for the dying patient: a critical analysis of its rise, demise and legacy in England. Wellcome Open Research.

[29] Bennett M, Ziegler L, Allsop M, al., et. (2016). What determines duration of palliative care before death for patients with advanced disease?. BMJ Open.

[30] Knight T, Malyon A, Fritz Z (2020). Advance care planning in patients referred to hospital for acute medical care: results of a national day of care survey. EClinicalMedicine.

[31] Lund S, Richardson A, May C (2015). Barriers to advance care planning at the end of life: an explanatory systematic review of implementation studie. PLoS One.

[32] Tanaka M, Kodama S, Lee I (2020). Forgoing life-sustaining treatment – a comparative analysis of regulations in Japan, Korea, Taiwan, and England. BMC Med Ethics.

[33] Uemura K, Iguchi A (1999). "euthanasia" and "dying with dignity": a legal point of view. Bioethics.

[34] Health and Global Policy Institute. 2018 Survey on Healthcare in Japan 2018. https://hgpi.org/en/research/hc-survey-2018.html. .

[35] Borgstrom E (2015). Social death in end-of-life care policy. Contemporary Social Science.

[36] Caswell G, Pollock K, Harwood R, Porock D (2015). Communication between family carers and health professionals about end-of-life care for older people in the acute hospital setting. BMC Palliative Care.

[37] Curry N, Castle-Clarke S, Hemmings N (2018). What can England learn from the long-term care system in Japan?.

[38] Carers UK. Unseen and undervalued: the value of unpaid care provided to date during the COVID-19 pandemic. 2020. https://www.carersuk.org/images/News_and_campaigns/Unseen_and_undervalued.pdf. .

[39] Morita T, Oyama Y, Cheng S (2015). Palliative care Physicians' attitudes toward patient autonomy and a good death in east Asian countries. J Pain Symptom Manag.

[40] Nakazawa E, Yamamoto K, Ozeki-Hayashi R (2019). Why Can’t Japanese people decide? — withdrawal of Ventilatory support in end-of-life scenarios and their indecisiveness. Asian Bioethics Review.

[41] National Cancer Center. The bereaved survey regarding patients care. Tokyo. 2020. https://www.ncc.go.jp/jp/cis/divisions/sup/project/090/about/index.html. Accessed 30 Oct 2021.

[42] Larsson H, Rämgård M, Bolmsjö I (2017). Older persons’ existential loneliness, as interpreted by their significant others - an interview study. BMC Geriatr.

[43] Bolmsjö I, Tengland P, Rämgård M (2019). Existential loneliness: an attempt at an analysis of the concept and the phenomenon. Nurs Ethics.

[44] Boston P, Bruce A, Schreiber R (2011). Existential suffering in the palliative care setting: an integrated literature review. J Pain Symptom Manag.

[45] Hasselaar J, Reuzel R (2007). Improving prescription in palliative sedation: compliance with Dutch guidelines. Arch Intern Med.

[46] Staudt C (2013). Whole-person, whole-community care at the end of life. Virtual Mentor.

[47] Morita T (2004). Palliative sedation to relieve psycho-existential suffering of terminally ill cancer patients. J Pain Symptom Manag.

[48] Murata H (2005). Spiritual pain and care in terminally ill patients: a phenomenological approach to clarification. Palliative Care (kanwa care).

[49] Murata H (2011). Spiritual pain and its care in patients with terminal cancer. J Japan Soc of Pain Clinicians.

[50] Miyashita M, Morita T, Sato K (2008). Good death inventory: a measure for evaluating good death from the bereaved family member's perspective. J Pain Symptom Manag.

[51] Chung B, Olofsson J, Wong F (2020). Overcoming existential loneliness: a cross-cultural study. BMC Geriatr.

